# IFN-*γ* Priming Effects on the Maintenance of Effector Memory CD4^+^ T Cells and on Phagocyte Function: Evidences from Infectious Diseases

**DOI:** 10.1155/2015/202816

**Published:** 2015-10-05

**Authors:** Henrique Borges da Silva, Raíssa Fonseca, José M. Alvarez, Maria Regina D'Império Lima

**Affiliations:** ^1^Departamento de Imunologia, Instituto de Ciências Biomédicas, Universidade de São Paulo, Avenida Prof. Lineu Prestes 1730, 05508-000 São Paulo, SP, Brazil; ^2^Department of Laboratory Medicine and Pathology, University of Minnesota, 2101 6th Street SE, Room 2-280, Minneapolis, MN 55414, USA

## Abstract

Although it has been established that effector memory CD4^+^ T cells play an important role in the protective immunity against chronic infections, little is known about the exact mechanisms responsible for their functioning and maintenance, as well as their effects on innate immune cells. Here we review recent data on the role of IFN-*γ* priming as a mechanism affecting both innate immune cells and effector memory CD4^+^ T cells. Suboptimal concentrations of IFN-*γ* are seemingly crucial for the optimization of innate immune cell functions (including phagocytosis and destruction of reminiscent pathogens), as well as for the survival and functioning of effector memory CD4^+^ T cells. Thus, IFN-*γ* priming can thus be considered an important bridge between innate and adaptive immunity.

## 1. Introduction

The immune system is continually exposed to a great diversity of pathogens. Among them, viruses, bacteria, protozoan parasites, and fungi present unique challenges for the host's immune system. In response to microorganisms, the adaptive immune system develops effector cells and functions capable of counteracting those threats. Among these effector cells, memory CD4^+^ T (T_M_) cells are considered a crucial population for the protective immunity against bacterial infections [1], viral infections [2], and protozoan infections such as malaria [3]. CD4^+^  T_M_ cells participate in the responses against secondary infections by potentiating antipathogen effector mechanisms of innate immunity [4], antibody production, and CD8^+^ T cell cytotoxicity [2].

In the past decades, however, it has become increasingly clear that the T_M_ population size is not a reliable marker of protective immunity per se. Zinkernagel and Hengartner previously argued that T_M_ cells could not provide protection against fast-dividing pathogens without the maintenance of highly responsive antigen-stimulated lymphocytes [5]. It was suggested that immunity, especially to chronic infection, is the combination of resting memory cells and activated effectors. The description of two distinct T_M_ cell subsets by Sallusto et al. [6] provides an additional basis for this hypothesis. Central memory T (T_CM_) cells and effector memory T (T_EM_) cells are classified based on their phenotype and their functional and trafficking characteristics [6, 7]. T_CM_ cells are defined by surface expression of CD62L and CCR7 molecules that allow these cells to circulate between secondary lymphoid tissues, entering the T cell zones [8]. In a T helper 1 (Th1) response, these cells produce IL-2 upon antigen reencounter and, later on, effector cytokines such as IFN-*γ*. T_EM_ cells, in contrast, have low expression of CD62L and CCR7 and migrate and localize into nonlymphoid, antigen-targeted tissues, where they are capable to quickly produce effector cytokines such as IFN-*γ* upon antigen reexposure [9, 10].

T_EM_ cells have been considered the predominant population elicited by chronic infections [1, 10]. Therefore, the knowledge about the T_EM_ cell origin, function, and survival is critical for vaccine development. In some infections, T_EM_ cells maintain increased effector function; however, this may require the continued presence of antigen, which can also lead to T cell exhaustion. Alternatively, in the absence of antigen, the T_CM_ population may remain expanded but without prompt functionality [11]. Among the possible mechanisms by which antigen persistence can drive the functioning of T_EM_ cells, the effects of IFN-*γ* cannot be underestimated. This cytokine, as cited above, is one of the main products secreted by T_EM_ cells in response to secondary antigen encounter [9], and its effects on both T_EM_ cells and the effector branch of the immune system are still to be completely understood. In this review, we describe recent data on the role of IFN-*γ* on the protective immunity to infectious diseases with a special focus on the importance of the IFN-*γ* priming.

## 2. The Concept of IFN Priming and Its Effects on Acute Infectious Diseases

The effects of IFN-*γ* on the immune system are diverse, and the importance of this cytokine on the functioning of innate immune cells has been previously discussed [19]. Dendritic cells and macrophages are tightly regulated by cytokines to rapidly respond to infections and also to avoid the undesirable effects of excessive activation. Suboptimal concentrations of IFN-*γ* do not actually activate these cells but make them prepared for a subsequent response to stimuli, which in excess can eventually cause deleterious consequences. This effect is denominated as IFN-*γ* priming and has been increasingly implicated in the immune response to several infectious diseases such as viral [20, 21], bacterial [15, 22], and parasitical [15] infections. The underlying molecular mechanism for IFN-*γ*-priming effect involves a complex network of IFN-inducible genes, mostly from the innate immune system [22], whose understanding is still limited [17]. It is presumed that IFN-*γ* priming induces posttranscriptional and/or epigenetic changes, which are responsible for subsequent Toll-like receptor (TLR) ligand-triggered inflammatory response and classical macrophage activation [20, 21, 23, 24]. Recently, it has been shown that IFN-*γ* priming downregulates the expression of miR-3473b, a microRNA that suppresses macrophage activation and inflammatory response through directly targeting phosphatase and tensin homolog (PTEN) and promoting IL-10 production [25]. Of note, IL-10 has been shown to prevent the development of immunopathology during acute malaria [26, 27], as well as in* Toxoplasma gondii* [28] and* Trypanosoma cruzi* [29] infections. However, IL-10 promotes pathogen survival by downregulating protective immune responses during infections with* Mycobacterium tuberculosis* [30],* Bordetella pertussis* [31], and human immunodeficiency virus (HIV) [32]. The dual role of IL-10 is exemplified in* Leishmania major* infection, where IL-10 from effector Th1 cells is required to control excessive inflammatory response during acute infection [33], but IL-10 from regulatory T cells contributes to parasite persistence by suppressing effector Th1 cells during chronic infection [34, 35].

The IFN-*γ* priming seems to be particularly involved in several aspects of the immune response to malaria. McCall et al. (2007) showed that* Plasmodium falciparum* induces enhanced responses to TLR agonists in peripheral blood mononuclear cells [36]. This notion was further corroborated by findings on human subjects and mice, both acutely infected with* P. falciparum* and* Plasmodium chabaudi*, respectively [37], which showed an increased innate immune response to unrelated pathogens, in a TLR- and IFN-*γ*-dependent manner. Besides the effect of IFN-*γ* priming on TLR signaling, TLR engagement seems to be necessary for the initial IFN-*γ* production, as described for rodent malaria [38, 39]. Thus, it is likely that TLR signaling mediates initial pathogen recognition, which in turn initiates early IFN-*γ* production that further boosts the innate response through TLR induction. This mechanism is supported by results with malaria—as previously described [37]—and with several other infections by pathogens such as* Listeria monocytogenes* [40],* L. major* [41],* Chlamydia pneumonia* [42],* T. cruzi* [43], and* Legionella pneumophila* [44].

In acute infectious diseases, the augmented gene expression of TLR-related molecules induced by IFN-*γ* likely favors the pathogen recognition by phagocytic cells. Thus, a primed innate immune system can be of utmost importance to prevent or limit aggressive infections, contributing to the host survival. In contrast, a possible deleterious effect of this hypersensitivity can be inferred from the enhanced susceptibility of* P. chabaudi*-infected mice to LPS treatment [37, 45]. This was in fact demonstrated by the enhanced susceptibility of IFN-*γ*-primed mice to bacterial sepsis, which showed increased TNF production upon LPS stimulation [46]. Higher sensitivity to secondary infections by bacteria, such as* Salmonella*, has also been observed in human malaria [47]. Moreover, this hyperactivation of the immune system may contribute for the posterior state of immune paralysis observed in septic patients [48].

CD4^+^ and CD8^+^ T cells are also responsive and can be primed by type I IFNs, IFN-*α*/*β*, which are produced virtually by any cell type after stimulation [49]. Type I IFNs can be produced in large amounts by myeloid cells upon bacterial infection [50, 51], or by plasmacytoid dendritic cells upon viral stimulation [52, 53]. Production of these cytokines occurs following pathogen recognition by Toll-like receptors (TLRs), RIG-I-like receptors (RLRs), NOD-like receptors (NLRs), and a growing family of intracellular DNA receptors, several of which promote signaling through stimulator of IFN genes (STING) [54, 55]. Similarly to IFN-*γ*, type I IFNs can drive preferentially the CD4^+^ T cell differentiation to Th1 phenotype by activating Signal Transducer and Activator of Transcription (STAT) proteins that increase the T cell response to IL-2 [56]. These cytokines also inhibit the Th2 development by epigenetic silencing of GATA3 gene regulatory regions [57]. Interestingly, type I IFN signaling inhibits IL-12 production [58, 59], which contrasts with the type I IFN effects on Th1 cell development. However, type I IFNs themselves also act as signal 3 cytokines during T cell activation—by promoting proliferation, survival, and effector cell differentiation [54]. Different from IFN-*γ*-induced priming that is always proinflammatory, the effects of type I IFNs on T cell function can be inhibitory in certain cases, especially when cytokine signaling precedes T cell receptor (TCR) engagement on T cells [54]. Optimal cross-priming of CD8^+^ T cells is seemingly dependent on type I IFN stimulation [60]. Furthermore, type I IFNs activate T cells and sensitize them to* Listeria*-induced apoptosis [61].

## 3. IFN-***γ*** Priming in Chronic Infections: Implications for Protective Immunity

IFN-*γ* is the main cytokine produced by T_EM_ cells committed for the Th1 phenotype (Th1_EM_ cells) upon infection by microorganisms, and it is believed to play a major role in the activation of innate immune response [62]. Thus, it is reasonable to imagine that these T_EM_ cells are a major source of the IFN-*γ* responsible for priming the innate immune cells during chronic infections, making it an important point of crosstalk between innate and adaptive immunity. Supporting this possibility, we have recently described that the presence of Th1_EM_ cells correlates with the continuous IFN-*γ* priming of innate immune cells during chronic malaria in mice [14]. This process is crucial for the protective immunity against reinfection with a heterologous strain of the parasite, which is not fully controlled by antibodies generated during primary infection with a different parasite clone. These findings help to explain why the immunity against* Plasmodium* is rapidly lost when the parasites are eliminated from the hosts, providing a molecular basis from strain-transcending immunity in human malaria [14, 63].

The innate immune effector mechanisms enhanced by IFN-*γ* priming are diverse, as pointed out by the high number of IFN-inducible genes upregulated in macrophages after* in vitro* IFN-*γ* priming [17, 64] or in mouse splenocytes during acute and chronic malaria [14, 37]. The biological significance of this priming is inferred from the genes expressed [14]. For instance, the upregulation of TLR-related and scavenger genes (such as CD36) possibly translates into an enhanced ability of innate immune cells to recognize and phagocytize circulating parasites, leading to an effective control of the disease [12, 45, 65]. It is important to note that an enhanced expression of TLRs facilitates the induction of the phagocytic program in innate immune cells. This was shown in chronic bacterial infections in which TLR3 and TLR9 expression leads to bacterial uptake by macrophages [13]. It is likely that IFN-*γ* secreted by Th1_EM_ cells also primes the innate immune cells during viral infections in a manner similar to that observed in malaria. A potent Th1_EM_ response is observed during infection with virus such as influenza [2, 66–68]. The IFN-*γ* priming of innate immune cells may ensure a rapid induction of the inflammatory response, as well as a state of refractoriness against viral proliferation in the surrounding tissues, which are important antiviral effector mechanisms [22, 69].

Besides busting proinflammatory responses, the IFN-*γ* priming associated with Th1_EM_ cells may trigger feedback inhibitory loops, such as those mediated by IL-10, STAT3, and Suppressor of Cytokine Signaling 1 (SOCS1) [17]. The increased transcription of* stat3* gene, another IFN-inducible gene, in mouse splenocytes from chronic malaria indicates a tight control of the innate immune system during continuous IFN-*γ* priming [14]. The STAT3 is a transcriptional activator of* Il10* gene, and a consequent effect of IL-10 production is induction of tolerance mediated by antigen-presenting cells (APCs) [70, 71]. This fine-tuned process seems to be a common feature of TLR-mediated immune responses, since TLR agonists induce a state of late immune tolerance through the inhibition of the corresponding signaling pathways [72, 73]. Of note, mice with chronic malaria display Th1_M_ cells coexpressing IFN-*γ* and IL-10, which are crucial for both the protective immunity to parasites and the protection against clinical manifestations of the disease [26]. A similar trend appears to happen in human malaria, since tolerance is often observed in patients from holoendemic areas [74]. The IFN-*γ* priming induced by Th1_EM_ cells, thus, appears to be a fundamental mechanism for an efficient, though tightly regulated, protective immunity against chronic infections.

## 4. IFN-***γ*** Priming Effects on** Th1**_**EM**_ Cells

The population of Th1_EM_ cells declines with time after infection in various experimental models of diseases caused by pathogens, such as* Plasmodium* [3],* Listeria* [75], and lymphocytic choriomeningitis virus (LCMV) [76, 77]. This observation suggests that the presence of prosurvival cytokines, such as IL-7 and IL-15, is not sufficient to maintain these cells [1]. On the other hand, large populations of specific Th1_EM_ cells usually persist for long periods of time during phagosomal infections, such as those caused by* Salmonella enterica* [78],* M. tuberculosis *[79, 80], and* L. major* [81]. Likewise, the presence of pathogens ensures the perpetuation of Th1_EM_ cells during polyomavirus infection [82] and malaria [3, 14]. Actually, the decline in the population of Th1_EM_ cells along with chronic malaria is related to the progressive control of residual parasitemia [3]. It has been shown that CD4^+^  T_EM_ cells have a rapid turnover in both human and mice [18, 83]. Thus, the continuous replenishment of this population may be induced by chronic infection, where pathogen antigens are available together with damage signals from injured tissues. In resume, the molecular signaling that is required for long-term persistence of Th1_EM_ cells seems to be present during active infection and rapidly disappear after its resolution. It is possible that antigen persistence and, consequently, TCR: MHC- (major histocompatibility complex-) peptide complex interactions play a role by itself in the maintenance of CD4^+^  T_M_ cells, and this has been a subject of interest for malaria [84] as well as for other infections [85, 86]. An interesting study on* Salmonella* infection in mice showed that peptide: MHC interaction in secondary lymphoid organs harboring bacteria for over 1 year after infection maintained the CD4^+^  T_M_ cell population stable [78]. IFN-*γ* priming might further potentiate these interactions—of note, the increased CD4^+^ T cell proliferation in response to* Plasmodium* parasites and parasite antigens indicates that IFN-*γ* priming enhanced antigen presentation during chronic malaria [14]. However, whether this effect was due to higher MHC class II (or costimulatory molecule) expression on APCs was not directly addressed and is still a matter of discussion.

The generation and maintenance of CD4^+^ and CD8^+^  T_EM_ cells are facilitated by strong TCR engagement [87, 88], but other signaling pathways may be implicated in these processes. The kinase mammalian target of rapamycin (mTOR) induces in CD8^+^ T cells a bias toward the glycolytic metabolism and the differentiation to effector functions [89, 90]. The STAT5-mediated IL-2 signaling pathway, a potent inhibitor of the Bcl-6 transcriptional factor and follicular T helper cell differentiation [91], promotes the expression of T-bet transcriptional factor in CD4^+^ T cells [75]. At this respect, it has been shown that Th1_EM_ cells have sustained expression of T-bet, both in humans [92] and mice [75]. T-bet upregulates IFN-*γ* production but is also an important target of IFN-*γ* signaling [93]. Therefore, IFN-*γ* is believed to induce—in conjunct with IL-12—IFN-*γ* production by Th1 cells. Thus, it is reasonable to hypothesize that IFN-*γ* plays a role in the generation and/or maintenance of Th1_EM_ cells during chronic infections.

We have recently addressed the effects of IFN-*γ* priming on Th1_EM_ cells in mice cured from chronic malaria in which the Th1_EM_ cell response rapidly declines [14]. In these cured mice, administration of suboptimal doses of IFN-*γ* leads to a shift from T_CM_ cells to T_EM_ cells and restores the proliferative and IFN-*γ* responses to parasites and TLR agonists. This effect could result from the rescue of cross-reactive T_CM_ cells driven by* Plasmodium*-unrelated antigens, a phenomenon previously described in human malaria [94]. However, in our study, the shift to T_EM_ cells was specifically observed in previously infected mice, pointing out to a preferential activity of IFN-*γ* priming on malaria-specific cells [14]. The exact pathways involved in the IFN-*γ* priming effects on the generation and/or maintenance of Th1_EM_ cells are still not well understood. It is likely that indirect signals derived from IFN-*γ*-primed innate immune cells play at least a partial role, and this is supported by the observation that TLR signaling is crucial for the maintenance of Th1_EM_ cells [14]. However, a direct effect of IFN-*γ* priming on Th1_EM_ cells cannot be excluded. Th1_EM_ cells express the IFN receptor (IFNR) on their surface, and IFN-*γ* signaling helps to maintain the Th1_EM_ phenotype [16]. The direct role of IFN-*γ* priming on Th1_EM_ cells induced during chronic malaria is currently under investigation by our research group. Preliminary results showed a requirement of IFNR expression on Th1_EM_ cells for their generation and maintenance (Borges da Silva, unpublished data).

Another infectious disease in which IFN-*γ* priming might be crucial for Th1_EM_ cells is tuberculosis. Evidences from human disease and experimental mouse models show that IFN-*γ* produced by CD4^+^ T cells is fundamental for* M. tuberculosis* control [95]. Importantly, expanded and sustained Th1 responses in the lungs are seemingly crucial for controlling chronic infection [96], making continuous IFN-*γ* priming possibly beneficial for bacterial clearance. However, in newborns vaccinated with Bacillus Calmette-Guérin (BCG) the IFN-*γ* production by Th1 cells did not correlate with disease protection [97].

In several infectious diseases, the pathogen persistence maintains short-lived effector T (T_EFF_) cells alongside T_EM_ cells. This seems to be particularly true for Th1-driving infections. Of note, T-bet^+^Ly6C^+^  T_EFF_ cells present during chronic* L. major* cutaneous infection seem to be crucial for the protective immunity against reinfection and are proposed as major contributors for the state of concomitant immunity observed during chronic infections [98]. It is especially important to consider that the expression of Ly6C in T_EFF_ cells is directly under the control of T-bet, which, as explained above, can be driven by IFN-*γ* [76, 93]. Thus, it would not be surprising if IFN-*γ* priming acts also directly on the maintenance of the T_EFF_ cells as well, especially considering the need for infection persistence for their survival [98].

## 5. Concluding Remarks

**Figure 1:**
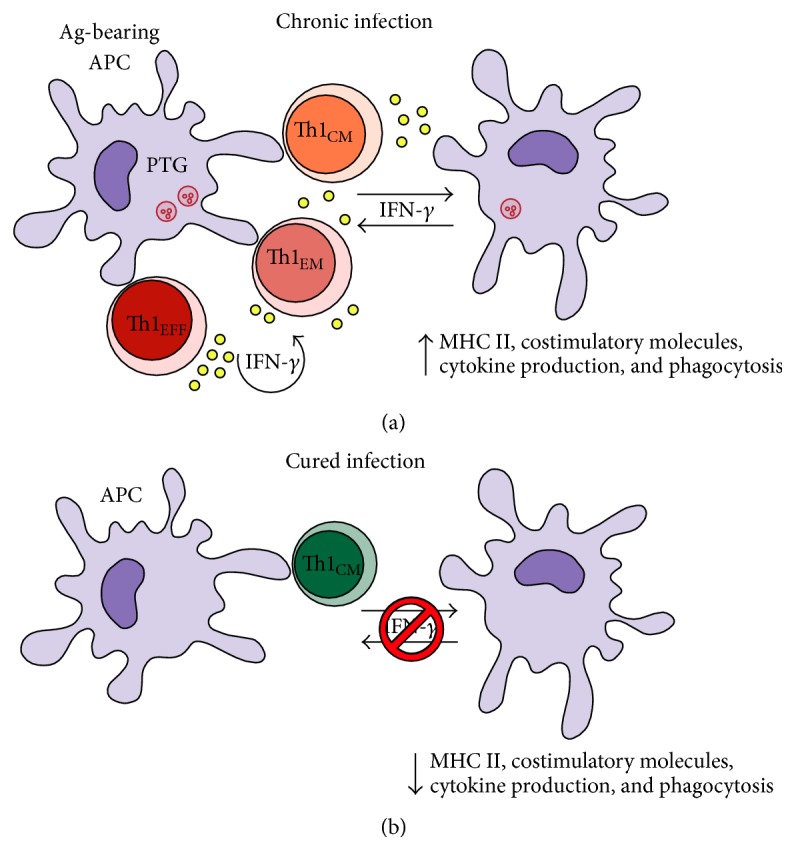
Schematic illustration to explain the IFN-*γ* priming effects on APCs and Th1_EFF_/Th1_EM_ cells during chronic infections. This figure explains how IFN-*γ* produced by Th1_EM_ cells act on APCs (usually DCs) and directly on CD4^+^ T cells during chronic infections. (a) When the pathogen is still present, antigen- (Ag-) bearing APCs activate CD4^+^ T cells that produce small amounts of IFN-*γ*. These small amounts of IFN-*γ* are enough to maintain APCs poised for function, for example, phagocytosis, cytokine production, and antigen presentation. At the same time, IFN-*γ* acts directly on CD4^+^ T cells and maintains the pool of Th1_EFF_/Th1_EM_ cells. Both effects culminate in enhanced immune system activation, cytokine production, and pathogen clearance. (b) After complete pathogen elimination, the IFN-*γ* priming on APCs and Th1_EFF_/Th1_EM_ cells ceases and, in consequence, these effector populations rapidly decline. The remaining Th1_CM_ cells are important to control a secondary infection. However, in some infectious diseases such as malaria, continuous IFN-*γ* priming, and persistence of Th1_EFF_/Th1_EM_ cells seem to be required to protect against reinfection.

The interplay between innate immune cells and Th1_EM_ cells during chronic infections is seemingly complex, involving a crosstalk between innate immune cells and Th1_EM_ cells. In this scenario, IFN-*γ* seems to play a crucial role as inducers of immune effector mechanisms in both sides, as exemplified in Table 1. Considering our current knowledge, the immune response to chronic infections might be defined as a circuit, where the two arms of the immune system (innate and acquired) constantly communicate with each other in order to achieve a tightly regulated, yet at most cases efficient, control of parasite load (Figure 1). To understand completely this relation, there is still the need to determine what all the “pieces in the puzzle” are, that is, to describe precisely all the aspects of the role of IFN-*γ* priming communication between T_M_ cells and the innate immune system. In the case of malaria, it will also be crucial to evaluate whether the observations in mouse models also hold true for humans, which are exposed to different degrees of reinfection with heterogeneous parasites. It is likely, though, that the importance of IFN-*γ* priming in strain-transcending immunity to malaria is a great starting point to explain, among other things, why it is so hard to achieve sterile immunity against* Plasmodium*; lowering the threshold for the activation of the host immune system could be a promising strategy for the improvement in the protective immunity against this parasite.

## Figures and Tables

**Table 1:** Effects of IFN-*γ* priming on innate immune cells and T_EM_ cells. The table summarizes the effects of IFN-*γ* priming on innate immune cells and on T_EM_ cells. The references relative to each function induced by priming are in parenthesis.

Innate immune cells	T_EM_ cells
↑ Phagocytosis [12, 13]	↑ Ag-specific proliferation [14]
↑ Antigen presentation [14, 15]	↑ T-bet expression [16]
↑ TLRs expression [14, 15, 17]	↑ Ag-driven IFN-*γ* production [14]
↓ Anergy [14, 15, 17]	↑ Population maintenance [14, 18]
